# Pharmacokinetics of oral and subcutaneous meloxicam: Effect on indicators of pain and inflammation after knife castration in weaned beef calves

**DOI:** 10.1371/journal.pone.0217518

**Published:** 2019-05-24

**Authors:** Daniela M. Meléndez, Sonia Marti, Edmond A. Pajor, Pritam K. Sidhu, Désirée Gellatly, Eugene D. Janzen, Timothy D. Schwinghamer, Johann F. Coetzee, Karen S. Schwartzkopf-Genswein

**Affiliations:** 1 Lethbridge Research Centre, Agriculture and Agri-Food Canada, Lethbridge, Alberta, Canada; 2 Department of Ruminant Production, IRTA, Caldes de Montbui, Barcelona, Spain; 3 Department of Production Animal Health, University of Calgary, Calgary, Alberta, Canada; 4 Department of Anatomy and Physiology, Kansas State University, Manhattan, KS, United States of America; University of Illinois, UNITED STATES

## Abstract

Oral meloxicam is labelled for reducing pain and inflammation associated with castration in cattle in Canada, however, subcutaneous meloxicam is only labelled for pain associated with dis-budding and abdominal surgery. The aim of this project was to determine the pharmacokinetic profile of oral (**PO**; 1.0 mg/kg BW) and subcutaneous meloxicam (**SC**; 0.5 mg/kg BW), and to assess the effect of meloxicam on physiological and behavioural indicators of pain associated with knife castration in 7–8 month old calves. Twenty-three Angus crossbred beef calves (328 ± 4.4 kg BW) were randomly assigned to two treatments: **PO** n = 12 or **SC** n = 11 administration of meloxicam immediately before knife castration. Physiological parameters included salivary and hair cortisol, substance P, haptoglobin, serum amyloid-A, weight, complete blood count, scrotal and rectal temperature. Behavioural parameters included standing and lying behaviour, pen behaviour and feeding behaviour. Data were analyzed using PROC GLIMMIX (SAS), with repeated measures using mixed procedures including treatment as a fixed effect and animal and pen as a random effect. The pharmacokinetic profile of the drug including area under the curve, volume of distribution and clearance was greater (*P <* 0.05) in PO than SC calves. After surgery, substance P concentrations, white blood cell counts (WBC), weight and lying duration were greater (*P <* 0.05) in PO than SC calves, while scrotal circumference was lower (*P <* 0.05) in PO calves than SC calves. Although statistical differences were observed for pharmacokinetic, physiological and behavioural parameters differences were small and may lack biological relevance.

## Introduction

Meloxicam is a non-steroidal anti-inflammatory drug (NSAID) that inhibits cyclooxygenase-2 (COX-2) enzymes which convert arachidonic acid into pro-inflammatory prostaglandins [1]. Meloxicam is approved for its use in cattle in the European Union and Canada, and it is an attractive analgesic option as it is effective following a single dose administration due to its long half-life in calves (subcutaneous: SC = 16.4 h; oral: PO = 27.5 h) [2,3]. In Canada, meloxicam is available for use in cattle in two presentations: meloxicam PO suspension (1.0 mg/kg) labelled for reducing pain and inflammation associated with band and knife castration and, injectable meloxicam (0.5 mg/kg) labelled as an adjuvant for diarrhea, mastitis, de-budding and abdominal surgery.

The Canadian Beef Codes of Practice [4] has set as a requirement the use of pain mitigation when performing painful husbandry procedures such as castration, spaying and dehorning. Castration is a routine practice which improves cattle management, avoids unwanted reproduction and increases meat quality [5]. Injectable meloxicam is not labelled for pain mitigation associated with castration however, previous studies have reported a reduction in physiological and behavioural indicators of pain in calves receiving SC meloxicam compared to un-medicated 1 week and 2 month old castrated calves [6,7]. Meloxicam tablets have been reported to decrease the inflammatory response in weaned calves after surgical castration [8,9], but no effects were observed in weaned calves after band castration [10]. The presentation of oral meloxicam used in the previous studies differs from the liquid formulation approved for use in cattle in Canada. Therefore, the aim of this study was to compare the pharmacokinetics (PK) of SC and PO meloxicam and to assess the effect of different routes of meloxicam administration on indicators of pain and inflammation in 7–8 month old calves during and after knife castration. We hypothesize that indicators of pain and inflammation will be mitigated after PO and SC administration but the effect will be observed at different time points after castration due to differences in PK.

## Materials and methods

This protocol was approved by the Animal Care Committee of Lethbridge Research and Development Centre (ACC number 1718). Animals were cared for in accordance with the Canadian Council of Animal Care guidelines [11].

### Animal housing and management

Twenty-three crossbred Angus beef calves of 328 ± 4.4 kg body weight (BW) and 7–8 months of age were used in a 28 day (d) experiment. Upon weaning, calves were vaccinated with Pyramid FP 5 (Pyramid FP 5, Boehringer Ingelheim (Canada) Ltd., Burlington, Ontario, Canada) and TASVAX (TASVAX, Merck Animal Health, Kirkland, Quebec, Canada) and housed in 4 experimental pens (5–6 calves/pen) for a 3 week adaptation period prior to the start of the trial. Pens (40.2 m × 27.4 m) contained straw bedding, *ad libitum* water provided through a centrally located water system and *ad libitum* feed consisting of a total mixed ration of 80% barley silage, 17% dry-rolled barley and 3% supplement with vitamins and minerals to meet beef cattle nutrition requirements [12].

Calves were equally distributed by weight into pens and randomly assigned to treatments. The day of castration, calves were restrained in a hydraulic squeeze chute (Cattlelac Cattle, Reg Cox Feedmixers Ltd, Lethbridge, Alberta, Canada) where they were sampled and castrated. The experiment consisted of two treatment groups: **PO**; n = 12 meloxicam (Solvet, Alberta Veterinary Laboratories, Calgary, Alberta, Canada) (1mg/kg BW) and **SC**; n = 11 meloxicam (Metacam 20 mg/mL, Boehringer Ingelhein, Burlington, Ontario, Canada) (0.5 mg/kg BW) administered immediately prior to knife castration. The same veterinarian performed the knife castration on all the calves by making a latero-lateral incision on the scrotum with a Newberry castration knife (Syrvet Inc., Waukee, IA) and an emasculator was used to crush and cut the spermatic cords.

### Sample collection

Sampling time points included 24 and 48 (h) prior to castration (d -1 and -2), immediately before castration (T0), as well as 30, 60, 90, 120, 150, 240 min and on d 1, 2, 3, 5, 7, 10, 14, 21 and 28 after castration.

#### Meloxicam

Meloxicam samples were collected on d -2, T0, 30, 60, 90, 120, 150, 240 min and on d 1, 2, 3, 5 and 7 after castration to determine plasma concentrations of meloxicam for all calves. Samples were collected into 6-mL lithium heparin tubes (BD vacutainer; Becton Dickinson Co., Franklin Lakes, NJ), centrifuged for 15 min at 1.5 × *g* at 4°C and the serum was stored at -80°C [13]. Samples were analyzed using high-pressure liquid chromatography (Agilent 1100 Pump, Column Compartment, and Autosampler, Santa Clara, CA, USA) with mass spectrometry detection (LTQ, Thermo Scientific, San Jose, CA, USA) at Iowa State University, College of Veterinary Medicine (Ames, IA).

The plasma concentration vs. time data of meloxicam following SC and PO meloxicam administration were analyzed to determine their PK profile using the software (Phoenix Win-Nonlin 7.0, Certara, Inc. Princeton, NJ, USA) as described in a previous study [2]. Non-compartment PK approach was applied to the data using a pre-structured model (Model: Plasma 200–202 with uniform weighting) in the software. The slope of terminal phase (λ_z_) of the log plasma concentration vs. time curve was estimated by means of linear regression; while the half-life of the terminal phase (λ_z-HL_) was calculated using the following equation: λ_z-HL_ = $\frac{0.693}{\lambda_{z}}$.

Area under the plasma concentration vs. time curve (AUC) and area under the first moment of the plasma concentration vs. time curve (AUMC) were calculated by use of the log- linear trapezoidal method [14]. Time range from the first measurement (T0) to the last measurement (d 7) of drug concentration was used for the calculation of AUC_0-last_ and AUMC_0-last_. The AUC and AUMC were extrapolated to infinity to determine AUC_0-∞_ and AUMC_0-∞_ to account for the total meloxicam exposure to calves [14]. Apparent volume of distribution during terminal phase (V_z_/F) and total systemic clearance scaled by bioavailability (CL/F) and mean residence time (MRT) of drug were also determined. Peak plasma concentration (C_max_) and time to achieve peak concentration (T_max_) were determined directly from the observed data.

#### Salivary cortisol

Salivary samples were collected on d -1, -2, T0, 30, 60, 90, 120, 150, 240 min and on d 1, 2, 3, 5, 7, 10, 14, 21 and 28 after castration. A cotton swab used to collect saliva from the oral cavity was stored in a plastic tube and frozen at– 20° C for further cortisol analysis [15]. Salivary cortisol concentrations were quantified using an enzyme immunoassay kit (Salimetrics, State College, PA). Inter-assay CV and intra-assay CV were 32.1% and 8.8%, respectively.

#### Hair cortisol

Hair from the forehead of the calves was clipped on d—2, 14 and 28 after castration. Samples were stored in plastic bags at room temperature and handled and analyzed as described by Moya et al. [16]. Cortisol was quantified using an enzyme-immunosorbent assay (Salimetrics, State College, PA). The intra-assay and the inter-assay’s CV were 8.8% and 11.0%, respectively.

### Substance P

Samples were collected from all calves through jugular venipuncture at d -1, -2, T0, 30, 60, 90, 120, 150 and 240 min, and on d 1, 2, 3, 5, 7, 10, 14, 21 and 28 after castration. Samples were collected and analyzed as previously described by Meléndez et al. [17]. Samples were collected into 6-mL tubes containing EDTA (BD vacutainer; Becton Dickinson Co., Franklin Lakes, NJ), where benzamidine hydrochloride was added to reduce substance P degradation and centrifuged for 15 min at 1.5 × *g* at 4°C and the serum was stored at -80°C. Samples were analyzed at Iowa State University, College of Veterinary Medicine (Ames, IA) with some modifications from the previously described procedure by Van Engen et al. [18]. The intra-assay CV was 8.8% and the inter-assay CV was calculated at 11.5%.

#### Haptoglobin and serum amyloid-A

Samples were collected from all calves through jugular venipuncture at d -1, T0, 90 and 240 min, and on d 1, 2, 3, 5, 7, 10, 14, 21 and 28 after castration. Blood samples were collected into 10-mL non-additive tubes (BD vacutainer; Becton Dickinson Co., Franklin Lakes, NJ), left at room temperature for 1 hour before being centrifuged for 15 min at 1.5 × *g* at 4°C and the serum was decanted and frozen at -80°C for further analysis [13]. The inter-assay CV for haptoglobin was 8.2%, while SAA intra-assay and inter-assay CV were 3.9% and 11.6%, respectively.

#### Complete blood cell count

Blood samples were collected through jugular venipuncture at d -2, -1, T0, 30, 60, 90, 120, 150, and 240 min, and on d 1, 2, 3, 5, 7, 10, 14, 21 and 28 after castration. Blood samples were collected into 6-mL EDTA tubes (BD vacutainer; Becton Dickinson Co., Franklin Lakes, NJ) and red blood cells and white blood cells were measured using a HemaTrueHematology Analyzer (Heska, Lobeland, Co).

#### Scrotal temperature

Images of the scrotum and its surrounding area were collected on d -2, -1, T0, 30, 60, 90, 120, 150, 240 min and on d 1, 2, 3, 5, 7, 10, 14, 21 and 28 after castration. A FLIR i60 infrared camera (FLIR Systems Ltd., Burlington, ON, Canada) was used to capture infrared images of the scrotal area at a distance of 1 m from the scrotum, and FLIR Tools version 5.1 (FLIR Systems Ltd.) was used to delineate the scrotal area and to record the maximum temperature [19]. An emissivity coefficient of 0.98 was used to analyze the images.

#### Scrotal circumference

The scrotum was evaluated on d -2, 90 and 240 min, and on d 1, 2, 3, 5, 7, 10, 14, 21 and 28 after castration using scrotal tape (Reliabull, Lane Manufacturing, Denver, CO) on the widest part of the scrotum [20].

#### Rectal temperature

A digital thermometer (M750 Livestock Thermometer, GLA Agricultural Electronics, San Luis Obispo, CA) was used to collect rectal temperature on d—2, -1, 0, 1, 2, 3, 5, 7, 10, 14, 21 and 28 after castration.

#### Weight

Calves were weighed in a hydraulic squeeze chute (Cattlelac Cattle, Reg Cox Feedmixers Ltd, Lethbridge, Alberta, Canada) on d -2, -1, T0, 1, 2, 3, 5, 7, 10, 14, 21 and 28 after castration.

#### Visual analog scale

Two experienced observers placed a mark along a 10 cm line (far left indicating no pain and far right extreme pain) as an indicator of their perception of the amount of pain calves were experiencing during castration [19]. Due to the experimental conditions observers were not blind to treatments.

#### Head movement

A video camera was placed in front of the head gate during castration to record head movement. An observer blind to treatment used the middle of the hairline of the muzzle as a reference point to track the distance (cm) of head movement during castration using Kinovea (General Public License) version 2 [13].

#### Chute movement

The movement of the animals in the chute during castration was quantified using strain gauges and accelerometers as previously described by Melendez et al. [13]. Briefly, the right and left head gate were equipped with strain gauges to measure the force cattle exerted on the head gate by pushing or pulling, while the chute was equipped with three 1-axis accelerometers (CXL-GP Series, Aceinna, Andover, MA) measuring lateral, vertical and horizontal movement. Analog signals (V) from the accelerometer and strain gauges were sent to a computer at a rate of 100 samples/s. Data from the accelerometers was added for each animal to obtain an overall acceleration force, and the data from the left and right head gate were added by animal to obtain an overall head gate force. Data from d -1 and d -2 was used as a baseline for each calf, this data was collected after the animal entered the chute and prior to sampling for a 20 second period. Variables included head gate and accelerometer number of peaks between 1 and 2 SD, 2 and 3 SD, and above or below 3 SD above and below the mean, and total area between the mean ± 1 SD, mean ± 2 SD, and mean ± 3 SD. These variables were divided by the time required to castrate each calf.

#### Pain sensitivity

Pain sensitivity was assessed as previously described by Marti et al. [20] using a Von Frey anesthesiometer (electronic von Frey anesthesiometer with rigid tip; 0 to 1000g; IITC-Life Science Instruments, Woodland Hills, California, USA) on the wound and on the skin adjacent to the wound. Animals were tested on d -2, -1, T0, 30, 90, 240 min and on d 1, 2, 3, 5, 7, 10, 14, 21 and 28 after castration while standing in the chute with their head restrained. The maximum pressure exerted on the wound before a behavioural reaction (steps, kicks or tail flicks) was recorded.

#### Stride length

Video recordings of calves walking through a 1 x 3 m alley were collected on d -2, -1, immediately after castration, 30, 60, 90, 120, 150, 240 min and on d 1, 2, 3, 5, 7, 10, 14, 21 and 28 after castration. Stride length was collected as described by Currah et al. [21] however a grid background wasn’t used and the image analysis software differed between studies. Observers blind to treatments took pictures of the back legs using GOM player (GOM Lab, Gretech Corporation, Seoul, South Korea) and Image J (National Institutes of Health Image, Bethesda, MD) was used to measure the stride distance (cm). Data from d 5 and 14 were removed from the analysis due to incomplete data for the majority of animals.

#### Pen behavior

An experienced observer blind to the treatments scored behaviour for a 4 hour period between 5 to 9 hour relative to castration on d 0 when calves returned to their home pen, and at the same time of the day on d 1, 2, 3 and 7 after castration. Focal animal sampling from continuous recordings [22] were used to score frequency of tail flicks, foot stamping, head turning and lesion licking and duration of standing, lying, walking and eating. Behaviours were modified from the ethogram described by Molony et al. [23]. Behaviours were defined as: a) eating: ingesting hay or straw from the ground or the feeder, b) lying: either lateral (laying with hip and shoulder on the ground with at least 3 limbs extended) or ventral (laying in sternal recumbency with legs folded under the body or one hind or front leg extended) lying, c) walking: walking forward more than 2 steps, d) standing: standing on all four legs, e) foot stamping: hind legs are lifted and forcefully placed on the ground or kicked outwards while standing, f) head turning: head is turned and touches the side of the calf’s body when standing, including head turning to groom, g) tail flicking: forceful tail movement beyond the widest part of the rump when standing, movement to one side is counted as one action, h) lesion licking: head turning to lick the lesion caused by castration while standing [17]. Intra-rater reliability was 0.98 respectively.

#### Standing and lying behavior

Accelerometers (Hobo pendant G, Onset Computer Corporation, Bourne, MA) were placed on the calves using Vet Wrap (Professional Preference, Calgary, Canada) to determine daily standing and lying percentage, and daily average standing and lying bout duration [24]. Accelerometers were wrapped in plastic to protect the device from moisture and in foam to avoid discomfort when placed above the hock [17]. Accelerometers were placed on d -1 and changed weekly to avoid inflammation of the area. Information from days when accelerometers were changed (d 7, 14, 21 and 28) were excluded from the analysis due to incomplete data collection.

#### Feeding behavior

The GrowSafe feed bunk monitoring system (GrowSafe Systems, Airdrie, Alberta, Canada) was used to record feeding behaviour. Each calf was fitted with a radio frequency ear tag and each pen was equipped with 5 feeding tubs which recorded feeding behaviour for each individual calf 24 hours a day over a 28 d period. Feeding duration (min/d), dry matter intake (kg/day), feeding rate (g/min), meal frequency (number/d), meal duration (min/meal) and meal size (kg/meal) were calculated from the feeding behaviour data [15]. As in the previous study, a meal criterion of 300 s was selected as it has been previously used in cattle [25, 26].

### Statistical analysis

The normal distribution of the residual was not assumed and therefore the models were “generalized” (SAS PROC GLIMMIX). For each model, a distribution was selected from the exponential family of distributions based on the model fit statistics, i.e., the Bayesian information criterion. The models were “mixed” due to the inclusion of fixed (treatment, the experimental covariate, and linear, quadratic, and cubic effects of time) and random (pen and animal) factors. In each model a covariate was included. The values of the covariate were averages from d 2 and 1 before castration (or values from d 1 or 2 before castration). In some cases, the polynomial (cubic and quadratic) effects of time were not statistically significant and (in those cases) the polynomial components were not included in the models.

## Results and discussion

The PK of meloxicam following intravenous (IV) or PO administration have been previously reported for cattle, sheep, goats, llamas and horses [3,27–32]. In the European Union and Canada, meloxicam has been approved for intramuscular (IM) and SC (0.5 mg/kg) use in cattle, as an adjunct therapy during the treatment of acute mastitis, diarrhea, respiratory disease and dehorning. In Canada PO meloxicam (1 mg/kg) has been approved for its use in cattle to mitigate pain associated with band and knife castration. The PK data are clinically useful as the terminal half-life of PO meloxicam at a dose of 1.0 mg/kg suggested that once a day administration provides analgesic efficacy in calves [3]. The PO route of drug administration is convenient, non-invasive, typically painless, and formulations are generally cheaper. Limitations of PO administration include a prolonged time of onset of analgesia after administration and unpredictable absorption due to varying gastric conditions and first pass hepatic biotransformation [33]. In contrast, SC administration offers the advantage of faster absorption and ease of administration. To our knowledge, there is only one previous study assessing PK following SC administration of meloxicam in cattle [2]. Therefore, the goals of this study were to describe the PK characteristics of meloxicam following SC administration to compare the pharmacokinetics of meloxicam after SC (0.5 mg/kg) and PO (1 mg/kg) administration. These data are important to optimize drug administration relative to the timing of the procedure and to design effective analgesic protocols for use in calves at the time of castration.

The time to reach peak plasma drug concentrations (T_max_ = 24.0 hour, PO; T_max_ = 3.7 hour, SC) after drug administrations differed (*P* ≤ 0.05) between treatments while no differences (*P* ≥ 0.10) were observed for peak plasma drug concentration (C_max_ = 2.32 μg/mL, PO; C_max_ = 2.37 μg/mL, SC) (Table 1). These findings were expected due to the differences in route of drug administration which have an effect on drug absorption. Similar T_max_ and C_max_ values were observed in calves receiving SC meloxicam with or without a lidocaine ring block prior to knife castration [2]. Similar findings were also reported in goats, where SC meloxicam administration had a significantly shorter T_max_ (3.20 hour) compared to PO meloxicam administration (14.3 hour) [31]. In contrast, mean C_max_ following SC meloxicam administration in the present study was higher than the value (C_max_ = 1.91 μg/mL) obtained for goats [31], while a lower C_max_ was observed following PO administration in comparison to the C_max_ (3.10 μg/mL) previously reported in calves [3]. Difference in age and breed of animals, in addition to the time of drug administration relative to the feeding regimen may be the reason for the discrepancies observed between studies.

**Table 1:** Mean ± SD PK parameters of meloxicam following PO (1mg/kg) and SC (0.5 mg/kg) administration in calves (n = 12).

Item	PO	SC	*P*-Value
*λ_z,_ 1/h	*0.045 ± 0.006	*0.043 ± 0.007	0.39
*λ_z_-HL	*15.6 ± 2.33	*16.2 ± 2.48	0.39
T_max_, h	24.0^a^ ± 0.00	3.7^b^ ± 0.72	<0.01
C_max,_ ng/ml	2325 ± 431.4	2374 ± 384.0	0.90
Cl_F, mL/h/kg	11.11^a^ ± 3.10	7.98^b^ ± 1.436	<0.01
AUC_0-24h,_ h × ng/mL	34195 ± 5493.9	39285 ± 6083.1	0.20
AUC_0-last,_ h × ng/mL	94992^a^ ± 20718.7	64320^b^ ± 11275.5	<0.01
AUC _0-∞,_ h × ng/mL	95160^a^ ± 20755.3	64455^b^ ± 11331.9	<0.01
AUC extrapolated, %	0.18^b^ ± 0.144	0.21^a^ ± 0.156	<0.01
AUMC_0-∞,_ h^2^ × ng/mL	3294678^a^ ± 956551	1483639^b^ ± 480681	<0.01
MRT0-∞, h	34.1^a^ ± 3.32	22.6^b^ ± 4.26	<0.01
Vz_F, mL/kg	244^a^ ± 43.2	183.5^b^ ± 21.85	<0.01

PK parameters were determined using non-compartment modeling.

*Harmonic means and rest of the means are geometric means ± SD.

^a-b^ Values with differing superscripts differ *P* <0.05.

The area under the curve (AUC = 95.16 μg × h/mL), V_z_/F = 244 mL/kg and Cl/F = 11.11 mL/h/kg were greater (*P* ≤ 0.05) in the calves receiving PO compared to SC meloxicam administration. The AUC is an indicator of the total drug exposure and it is dependent on dose and rate of elimination. Calves given PO meloxicam received a higher dose (1 mg/kg) compared to SC meloxicam administration (0.5 mg/kg). In general the oral dose of a particular drug is higher than the dose of the injectable formulation due to the metabolism that occurs in the gastrointestinal wall and the liver which is commonly known as *first pass effect*. A higher dose of meloxicam given to the PO calves seems to be the major contributing factor for a greater AUC as the elimination rate (λ_z =_ 0.043–0.045 1/h) is approximately the same for both treatment groups.

The SC calves had lower (*P* ≤ 0.05) clearance (Cl/F = 7.98 mL/h/kg) of meloxicam than the PO calves, which is in agreement with the longer elimination half-life (λ_z_-HL = 16.2 h) of the SC treatment than the PO (15.2 h) administration. The λ_z_-HL (16.2 h) in calves was slightly higher than that reported for goats (15.1 h) after SC meloxicam administration using the same dose of 0.5 mg/kg [31], while higher values for λ_z_-HL (27.5 h) and AUC (164.4 μg.h/mL) have been reported following PO administration of meloxicam in calves [3]. In the previously mentioned trial PK analysis showed that the AUC extrapolation range was 23.0–39.4% in four calves and 4.14–5.85% in two calves. In contrast, PK analysis for the current study was done with AUC extrapolation of 0.18%. In addition, there was a difference in the sampling schedule design between the two studies. In the present study, blood samples for meloxicam determination were collected for 168 hours after drug administration, however, in the previous study blood samples were collected up to 96 hours post drug administration. Insufficient sampling times in the descending part of the curve may lead to overestimation of AUC [34]. This could be the reason for a greater AUC in the previous study compared to the AUC obtained in the current study. The AUC in calves was greater than the AUC reported in sheep (75.09 μg × h/mL) [30] and goats (23.24 μg × h/mL) [29], indicating that meloxicam is eliminated at a slower rate in calves than small ruminant species.

The limited V_z_/F = 244 mL/kg observed in the present study after PO meloxicam administration is similar to previous values reported for calves (242 mL/kg) [3] and sheep (293 mL/kg) [30]. A low V_z_/F indicates that the drug is mainly found in the vascular space as opposed to the extravascular space. Meloxicam is a drug which is highly bound to plasma proteins and its molecules are ionized at physiological pH in ruminants, therefore, it is mainly found in the vascular space. This finding is in accordance with a previous study reporting limited volumes of distribution in ruminants receiving NSAIDs [35].

Although differences were observed between PO and SC treatments further PK studies are needed to evaluate if these differences are biologically relevant. However, low values of clearance and a terminal half-life of 16.2 hours following SC administration suggests that once a day dosing might prove effective to maintain analgesic effect in calves. Pharmacodynamic studies demonstrating the efficacy of SC meloxicam at this dose are required for future indication of SC meloxicam in calves.

Substance P is a neuropeptide associated with the modulation of pain, stress and anxiety [36]. During tissue injury and inflammation the pain threshold can be reduced by the effect of pro-inflammatory substances, such as prostaglandin E_2_ which has the ability to stimulate the release of substance P from sensory neurons, therefore increasing the sensitivity of sensory neurons to physical or chemical stimuli [37]. A previous study identified substance P as a potentially useful biomarker of pain when greater substance P concentrations were reported in surgically castrated calves (506.4 ± 38.11 pg/mL) compared to un-castrated (386.4 ± 40.09 pg/mL) 4 to 6 month old calves, while no differences were observed in plasma cortisol concentrations [38]. Contrary to the previous findings, several studies have reported lack of differences in substance P concentrations after different methods of castration in calves of different ages [6,10,17,39]. In the present study substance P concentrations were greater (*P* ≤ 0.05) in PO than SC calves (Table 2). Based on the limits of 95% confidence, PO calves are expected to have 1 to 9% higher substance P concentrations than SC calves. Meloxicam has been previously reported to decrease substance P concentrations in the case of acute synovitis in the horse, as well as dehorning and castration in cattle [7,40,41]. The differences observed between treatments could be attributed to a faster absorption rate which could inhibit the release of inflammatory substances sooner in the inflammatory cascade, therefore affecting overall substance P concentrations. Although statistically significant, differences observed between treatments were very small (4.24 pg/mL) in comparison to the difference observed in the previous study (120 pg/mL) [38]. Caution should be taken when comparing results as age and sampling time points differ between studies. To our knowledge there are no studies assessing the effect of route of drug administration on substance P concentrations.

**Table 2:** Least square means (± SEM) of physiological parameters minutes and days after castration of surgical castrated weaned Angus crossbred calves receiving PO or SC meloxicam^1^.

	Treatment (T)^2^				*P*-value
Item, units	PO	SC	SEM^3^	T	Time
Salivary Cortisol, nmol/L	3.0	3.1	0.07	0.64	<0.01
Hair Cortisol, nmol/L	2.1	2.3	0.10	0.44	0.79
Substance P, pg/mL	83.0^a^	78.7^b^	0.02	0.01	0.01
Haptoglobin, g/L	0.6	0.7	0.08	0.24	<0.01
Serum amyloid-A, ug/mL	64.5	75.4	0.10	0.10	<0.01
CBC					
WBC, 10^9^/L	10.7^a^	10.1^b^	0.01	<0.01	<0.01
RBC, 10^12^/L	7.2	7.3	0.002	0.06	<0.01
Scrotal temperature,°C	33.9	33.8	0.01	0.33	0.09
Rectal temperature,°C	39.4	39.4	0.001	0.66	0.01
Scrotal circumference, cm	24.8^b^	26.1^a^	0.00004	0.01	<0.01
Weight, kg	326.6^b^	327.2^a^	0.001	<0.01	0.16

^1^Values in the table represent the mean of T0, 30, 60, 90, 120, 150 and 240 min and day 1, 2, 3,5,10,14, 21 and 28 after castration for salivary cortisol, substance P, scrotal temperature and CBC; the mean of day 14 and 28 after castration for hair cortisol; the mean of T0, 90 and 240 min and day 1, 2, 3, 5, 7, 10, 14, 21 and 28 after castration for SAA and haptoglobin; the mean of T0, 1, 2, 3, 5, 7, 10, 14 and 28 after castration for rectal temperature and weight; the mean of 90 and 240 min and day 1, 2, 3, 5, 7, 10, 14, 21 and 28 after castration for scrotal circumference.

^2^ Treatments administered immediately prior to castration: PO: oral meloxicam; SC: subcutaneous meloxicam.

^3^The values correspond to non-transformed means, however, the SEM and the *P-*values correspond to GLIMMIX analysis using napierian log transformation.

^a-b^ Values with differing superscripts differ *P* <0.05.

WBC counts were greater (*P* ≤ 0.05) in PO than SC calves. Based on the limits 95% confidence, PO calves are expected to have 2 to 9% higher WBC counts than SC calves. Castrated calves have been previously reported to have greater WBC counts than sham castrated calves [8,42,43] and meloxicam has been previously reported to decrease the WBC counts after castration in 1 week, 2 month and weaned calves [2,6–8]. Similar to the results observed for substance P, it is likely that SC calves had lower WBC counts due to a faster onset of action. Although differences were observed between treatments, the WBC count was within the normal range (WBC: 4–12 × 10^3^/μL) [44].

Weight was lower (*P* ≤ 0.05) in PO than SC calves. Based on the limits 95% confidence, PO calves are expected to have 0.25 to 0.08% lower weight than SC calves. Weight was assessed in the present study as an indicator of welfare as animals that are in pain generally reduce feed consumption which could potentially affect their average daily gain (ADG). Previous studies assessing the effect of castration in beef cattle have reported a decrease in ADG after knife and band castration, but performance parameters were not affected by medication [10,15,19,45]. These findings are similar to the results observed for substance P and WBC counts. If an analgesic and anti-inflammatory effect is achieved sooner, it is more likely that calves will be willing to walk to the feed bunk and eat sooner which could potentially affect weight gain.

Scrotal circumference was lower (*P* ≤ 0.05) in PO than SC calves. In the present study, scrotal circumference was assessed as an indicator of inflammation. Previous studies have reported an increase in scrotal circumference after band [42], knife [46], and burdizzo [47] castration in cattle. A previous study reported that the combination of lidocaine and meloxicam was more effective at reducing scrotal circumference than meloxicam alone [2]. The result for scrotal circumference is contrary to the results observed for substance P, WBC and weight. A possible explanation for the reduction in scrotal inflammation observed in PO calves could be due to the greater exposure to meloxicam as PO calves had greater AUC than SC calves.

No differences were observed in behaviour during castration (Table 3) and no differences were observed for behaviour after castration with the exception of lying and standing (Table 4). Lying percentage was greater (*P* ≤ 0.05) in PO than SC calves, while SC calves had greater (*P* ≤ 0.05) standing duration than PO calves. Previous studies have reported an increase in standing duration after castration in comparison to prior to castration [48]. Similar results have reported greater standing duration in knife castrated calves compared to band and control 2 month and 4 month old calves [17] suggesting that lying behaviour could be associated with comfort. Meloxicam treated calves had greater lying duration than non-medicated calves after knife castration and the combination of knife castration and branding [6]. Similar studies assessing the effect of meloxicam after a painful procedure reported greater lying duration in cattle after a C-section [49] and after dehorning [50] when compared to the placebo group. The effect of painful procedures and medication on lying behaviour support the notion of lying as a comfort indicator.

**Table 3:** Least square means (±SEM) of behavioural parameters during castration of surgical castrated weaned Angus crossbred calves receiving PO or SC meloxicam^1^.

	Treatment (T)^2^		*P*-value	
Item, units	PO	SC	SEM^3^	T
VAS, cm	3.8	3.6	0.18	0.75
Leg movements, n	13.6	14.7	0.08	0.42
Head movement, cm	2734	2391	0.11	0.35
*Accelerometers*				
Peaks between ± 1–2 SD, n	174	186	38.0	0.83
Peaks between ± 2–3 SD, n	72	49	24.9	0.43
Peaks above and below 3 SD, n	57	44	13.7	0.53
TA above and below 1 SD, V × s	7.0	5.3	0.20	0.08
TA above and below 2 SD, V × s	4.3	2.9	0.03	0.13
TA above and below 3SD, V × s	3.2	2.0	0.07	0.11
*Strain Gauges*				
Peaks between ± 1–2 SD, n	138	302	79.2	0.12
Peaks between ± 2–3 SD, n	177	65	24.9	0.10
Peaks above and below 3 SD, n	413.0	370.1	117.6	0.76
TA above and below 1 SD, V × s	210.9	260.9	0.17	0.83
TA above and below 2 SD, V × s	132.2	165.9	0.47	0.24
TA above and below 3SD, V × s	89.2	122.3	0.42	0.79

^1^Values in the table represent the mean of VAS, leg movement, head movement and chute behaviour assessed at the time of castration.

^2^ Treatments administered immediately prior to castration: PO: oral meloxicam; SC: subcutaneous meloxicam.

^3^Values in the table correspond to non-transformed means; however, SEM and *P*-values correspond to the scale of inference (distribution of SAS PROC GLIMMIX), analysis using square root + 1 transformed data for VAS, leg movement, head movement and chute behaviour.

**Table 4:** Least square means (± SEM) of behavioural parameters minutes and days after castration of surgical castrated weaned Angus crossbred calves receiving PO or SC meloxicam^1^.

	Treatment (T)^2^				*P*-value
Item, units	PO	SC	SEM^3^	T	Time
Von Frey, g	324.2	342.0	0.00	0.65	<0.01
Stride length, cm	52.9	53.2	0.02	0.64	0.02
*Pen behaviour*					
Lying, min	65.2	53.1	0.76	0.29	0.02
Standing, min	104.4^b^	114.6^a^	0.00	0.05	0.05
Walking, min	10.4	12.3	0.16	0.31	<0.01
Eating, min	35.4	29.6	0.21	0.72	0.20
Tail flick, n	1064.9	1013.0	0.38	0.45	0.60
Foot stamp, n	4.1	5.6	0.34	0.92	0.36
Head turning, n	5.9	10.1	0.00	0.10	0.02
Lesion licking, n	1.4	2.3	0.06	0.08	0.02
*Standing/Lying behaviour*					
Standing duration, min	64.1	70.2	0.06	0.06	0.00
Lying duration, min	67.6	69.8	0.00	0.39	<0.01
Standing time, %	41.7	42.9	0.04	0.13	0.01
Lying time, %	55.3^a^	52.9^b^	0.02	0.04	<0.01
*Feeding behaviour*					
Dry matter feed intake, kg/d	5.5	5.6	0.10	0.88	<0.01
Feeding time, min/d	134	134	0.00	0.97	<0.01
Feeding rate, g/min	43.0	43.7	0.02	0.45	0.13
Meal frequency, meal/d	11.9	11.8	0.02	0.68	0.88
Meal duration, min/meal	11.3	11.3	0.00	0.76	<0.01
Meal size, kg/meal	0.6	0.6	0.02	0.86	0.09

^1^Values in the table represent the mean of immediately after castration, 30, 60, 90, 120, 150, 240 min and day 1, 2, 3, 7, 10, 21 and 28 after castration for SL; the mean of d 0, 1, 2, 3 and 7 for pen behaviour; the means of d 0 to 28 after castration, excluding sampling days, for standing and lying behaviour; the means of d 0 to 28 for feeding behaviour.

^2^ Treatments administered immediately prior to castration: PO: oral meloxicam; SC: subcutaneous meloxicam.

^3^The values correspond to non-transformed means, however, the SEM and the *P-*values correspond to GLIMMIX analysis.

^a-b^Least square means within a row with differing superscripts differ (*P* ≤ 0.05)

If the differences observed in the present study were biologically relevant, both PO and SC meloxicam administration reduced pain and/or inflammation indicators. The greater exposure to meloxicam (AUC) observed can explain lower standing duration and scrotal inflammation, and the greater lying percentage observed in the PO calves. On the other hand, the T_max_ could explain the lower substance P concentrations, WBC counts and weight, as a faster onset of action from SC meloxicam administration could inhibit the production of pro-inflammatory substances sooner in the inflammatory process which could consequently lead to a reduced magnitude of inflammation. In addition, SC calves were likely to reach therapeutic meloxicam concentrations sooner than PO calves, therefore a faster analgesic and anti-inflammatory effect could motivate calves to eat sooner after the procedure which could explain the difference observed for weight. Differences observed between treatments for substance P (4.24 pg/mL), WBC (0.05 × 10^9^), weight (0.54 kg), scrotal circumference (1.24 cm) lying (2.85%) and standing (10.2 min) are relatively small and although statistically significant these results may lack biological relevance. Lack of differences observed in the rest of parameters could be due to a small sample size, lack of sensitivity of the parameters assessed or because in fact there were no differences between treatments. Previous studies assessing SC meloxicam have reported a reduction in indicators of pain and/or inflammation in medicated than un-medicated castrated beef calves [2,6,7], however, a limitation of the current study is lack of internal sensitivity due to the absence of a control group that did not receive pain control.

The purpose of this study was to assess the PK of PO and SC meloxicam and the effect of drug administration route on physiological and behavioural indicators of pain. Although statistical differences were observed in PK, physiological and behavioural parameters, differences observed may lack biological relevance. Based on these results few differences were observed in physiological and behavioural indicators of pain after PO and SC meloxicam during and after castration in 7–8 month old beef calves. Further studies are needed to determine if the differences observed are biologically relevant.

## Supporting information

S1 Dataset (XLSX)

S1 File (DOCX)
