# Supplementary material for: Pharmacokinetics of oral and subcutaneous meloxicam: Effect on indicators of pain and inflammation after knife castration in weaned beef calves
Source: PLoS One. 2019 May 24;14(5):e0217518. doi: 10.1371/journal.pone.0217518 (PMC6534336; doi:10.1371/journal.pone.0217518)
Supplement: S1 File — (DOCX) [file pone.0217518.s002.docx]

# Supporting information

S1 Figure. Panel of nonparametric LOESS regressions of the predicted values of (a) substance P, (b) weight, (c) scrotal circumference, (d) the natural log of WBC, (e) and lying (solid lines) over time (predicted by generalized linear mixed models). Dashed lines indicate upper and lower limits (95% confidence). Hollow circles indicate observed values.

(a)


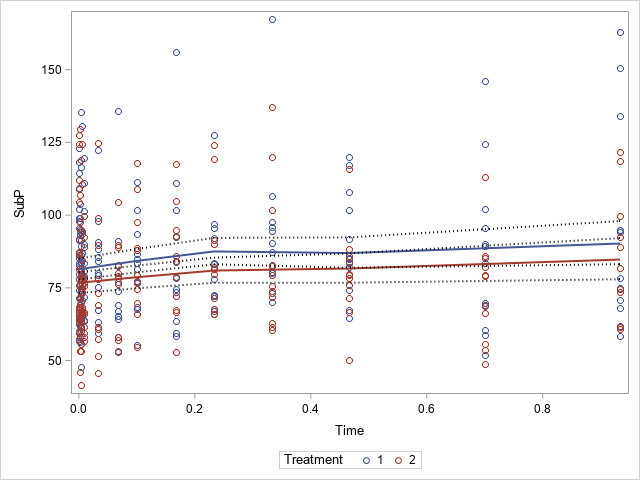


(b)


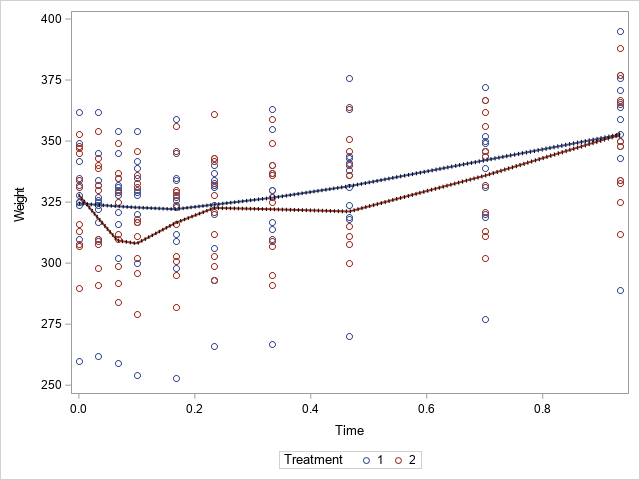


(c)


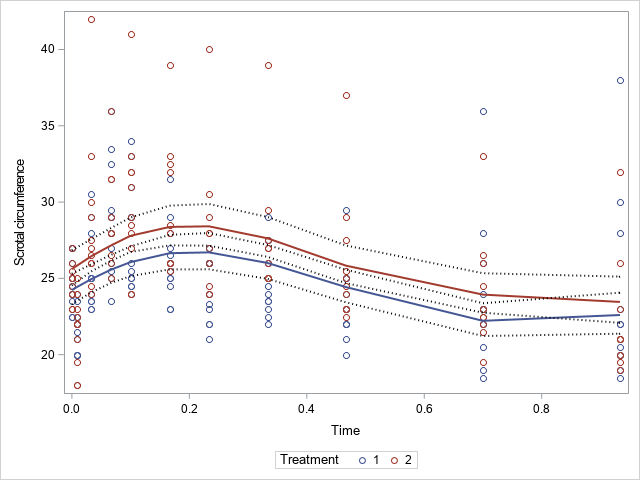


(d)


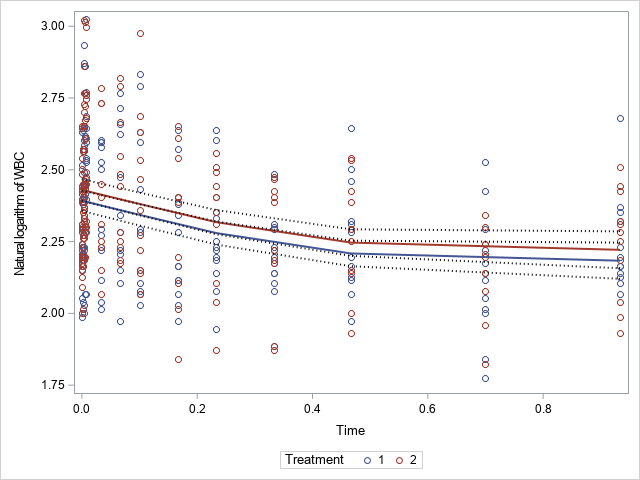


(e)


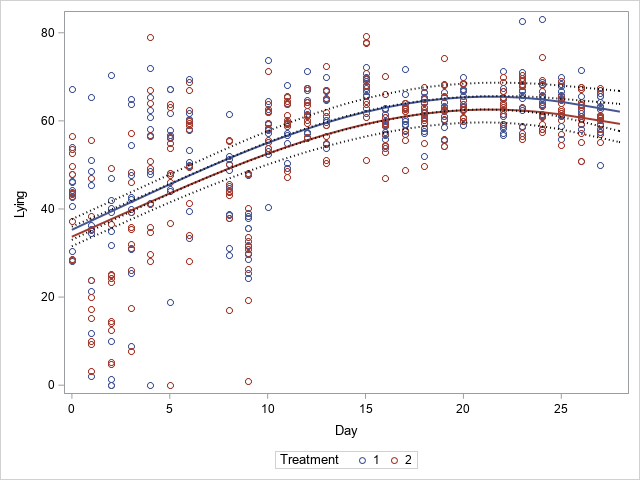


S2 Table. Generalized linear mixed modelling indicating the statistical significance of the fixed factors: treatment, covariate and the linear, quadratic, and cubic effects of time.

|  | **Table 3. Type III tests of fixed effects** | | | | | | |  |
| --- | --- | --- | --- | --- | --- | --- | --- | --- |
| Experimental variable | DIST = | Link Function | Hypotheses tests (*F*-value, *p*-value) | | | | | |
|  |  |  | Treatment | Time |  |  | Covariate | |
|  |  |  |  | linear | quadratic | cubic |  | |
| Haptoglobin | T | Identity | *F*_1,266_ = 1.41, *p* = 0.2361 | *F*_1,266_ = 60.94, *p* < .0001 | *F*_1,266_ = 73.48, *p* < .0001 | *F*_1,266_ = 67.61, *p* < .0001 | *F*_1,266_ = 0.56, *p* = 0.4553 | |
| SAA | LOGN | Identity | *F*_1,266_ = 2.72, *p* = 0.1002 | *F*_1,266_ = 30.18, *p* < .0001 | *F*_1,266_ = 40.81, *p* < .0001 | *F*_1,266_ = 36.23, *p* < .0001 | *F*_1,266_ = 16.19, *p* < .0001 | |
| Cortisol | GAMMA | Log | *F*_1,292_ = 0.22, *p* = 0.6397 | *F*_1,292_ = 111.41, *p* < .0001 | *F*_1,292_ = 58.89, *p* < .0001 | *F*_1,292_ = 38.94, *p* < .0001 | *F*_1,292_ = 27.81, *p* < .0001 | |
| Hair Cortisol | LOGN | Identity | *F*_1,32_ = 0.61, *p* = 0.4393 | *F*_1,32_ = 0.07, *p* = 0.7898 | . | . | *F*_1,32_ = 0.80, *p* = 0.3763 | |
| Substance P | GAMMA | Log | *F*_1,354_ = 7.46, *p* = 0.0066 | *F*_1,354_ = 7.86, *p* = 0.0053 | *F*_1,354_ = 4.91, *p* = 0.0274 | *F*_1,354_ = 4.17, *p* = 0.0420 | *F*_1,354_ = 129.37, *p* < .0001 | |
| Rectal temperature | LOGN | Identity | *F*_1,204_ = 0.19, *p* = 0.6639 | *F*_1,204_ = 6.37, *p* = 0.0123 | *F*_1,204_ = 0.57, *p* = 0.4492 | *F*_1,204_ = 6.85, *p* = 0.0095 | *F*_1,204_ = 1.39, *p* = 0.2401 | |
| Weight | GAMMA | Log | *F*_1,218_ = 15.81, *p* < .0001 | *F*_1,218_ = 2.03, *p* = 0.1558 | *F*_1,218_ = 8.66, *p* = 0.0036 | . | *F*_1,218_ = 113845, *p* < .0001 | |
| Scrotal Circumference | IG | Reciprocal Square | *F*_1,242_ = 6.90, *p* = 0.0092 | *F*_1,242_ = 18.08, *p* < .0001 | *F*_1,242_ = 18.66, *p* < .0001 | *F*_1,242_ = 14.46, *p* = 0.0002 | *F*_1,242_ = 12.18, *p* = 0.0006 | |
| VonFrey | IG | Reciprocal Square | *F*_1,286_ = 0.90, *p* = 0.3433 | *F*_1,286_ = 24.36, *p* < .0001 | *F*_1,286_ = 22.75, *p* < .0001 | *F*_1,286_ = 18.68, *p* < .0001 | *F*_1,286_ = 1.18, *p* = 0.2791 | |
| Stride Length | LOGN | Identity | *F*_1,312_ = 0.22, *p* = 0.6377 | *F*_1,312_ = 5.64, *p* = 0.0182 | . | . | *F*_1,312_ = 3.57, *p* = 0.0597 | |
| Scrotal temperature | GAMMA | Log | *F*_1,358_ = 0.96, *p* = 0.3274 | *F*_1,358_ = 2.81, *p* = 0.0946 | *F*_1,358_ = 2.97, *p* = 0.0856 | *F*_1,358_ = 9.47, *p* = 0.0023 | *F*_1,358_ = 16.70, *p* < .0001 | |
| RBC | IG | Reciprocal Square | *F*_1,119_ = 3.54, *p* = 0.0624 | *F*_1,119_ = 25.44, *p* < .0001 | *F*_1,119_ = 19.01, *p* < .0001 | . | *F*_1,119_ = 139.81, *p* < .0001 | |
| WBC | LOGN | Identity | *F*_1,357_ = 8.88, *p* = 0.0031 | *F*_1,357_ = 43.09, *p* < .0001 | *F*_1,357_ = 17.26, *p* < .0001 | . | *F*_1,357_ = 300.02, *p* < .0001 | |
| VAS | LOGN | Identity | *F*_1,40_ = 0.10, *p* = 0.7482 | . | . | . | . | |
| Leg movement | GAMMA | Log | *F*_1,40_ = 0.67, *p* = 0.4185 | . | . | . | . | |
| Feeding Time | IG | Reciprocal Square | *F*_1,652_ = 0.00, *p* = 0.9694 | *F*_1,652_ = 25.78, *p* < .0001 | *F*_1,652_ = 34.55, *p* < .0001 | *F*_1,652_ = 19.70, *p* < .001 | . | |
| Feeding Intake (DM) | IG | Reciprocal Square | *F*_1,652_ = 0.02, *p* = 0.8756 | *F*_1,652_ = 47.33, *p* < .0001 | *F*_1,652_ = 100.05, *p* < .0001 | *F*_1,652_ = 96.43, *p* < .0001 | . | |
| Feeding Rate | GAMMA | Log | *F*_1,652_ = 0.58, *p* = 0.4466 | *F*_1,652_ = 2.33, *p* = 0.1277 | *F*_1,652_ = 3.18, *p* = 0.0751 | *F*_1,652_ = 15.71, *p* < .0001 | . | |
| Meal Frequency | N | Log | *F*_1,652_ = 0.17, *p* = 0.6803 | *F*_1,652_ = 0.02, *p* = 0.8773 | *F*_1,652_ = 5.84, *p* = 0.0159 | *F*_1,652_ = 10.89, *p* = 0.0010 | . | |
| Meal Duration | IG | Reciprocal Square | *F*_1,651_ = 0.09, *p* = 0.7646 | *F*_1,651_ = 30.18, *p* < .0001 | *F*_1,651_ = 45.88, *p* < .0001 | *F*_1,651_ = 26.63, *p* < .0001 | . | |
| Meal Size | N | Identity | *F*_1,652_ = 0.03, *p* = 0.8618 | *F*_1,652_ = 2.97, *p* = 0.0851 | *F*_1,652_ = 41.13, *p* < .0001 | *F*_1,652_ = 60.58, *p* < .0001 | . | |
| Standing Duration | LOGN | Identity | *F*_1,544_ = 3.68, *p* = 0.0557 | *F*_1,544_ = 11.03, *p* = 0.0010 | *F*_1,544_ = 35.97, *p* < .0001 | *F*_1,544_ = 44.65, *p* < .0001 | . | |
| Lying Duration | IG | Reciprocal Square | *F*_1,543_ = 0.75, *p* = 0.3881 | *F*_1,543_ = 15.85, *p* < .0001 | *F*_1,543_ = 13.78, *p* = 0.0002 | *F*_1,543_ = 10.87, *p* = 0.0010 | . | |
| Standing percentage | GAMMA | Log | *F*_1,553_ = 2.33, *p* = 0.1273 | *F*_1,553_ = 6.48, *p* = 0.0112 | *F*_1,553_ = 3.06, *p* = 0.0809 | *F*_1,553_ = 9.72, *p* = 0.0019 |  | |
| Lying percentage | GAMMA | Log | *F*_1,552_ = 4.18, *p* = 0.0413 | *F*_1,552_ = 138.28, *p* < .0001 | *F*_1,552_ = 59.55, *p* < .0001 | . | . | |
| Head Movement | LOGN | Identity | *F*_1,18_ = 0.94, *p* = 0.3457 | . | . | . | . | |
| AcTotalArea1sd | GAMMA | Log | *F*_1,18_ = 3.38, *p* = 0.0827 | . | . | . | . | |
| AcTotalArea2sd | IG | Reciprocal Square | *F*_1,18_ = 2.52, *p* = 0.1297 | . | . | . | . | |
| AcTotalArea3sd | IG | Reciprocal Square | *F*_1,18_ = 2.88, *p* = 0.1070 | . | . | . | . | |
| StTotalArea1sd | LOGN | Identity | *F*_1,18_ = 0.05, *p* = 0.8315 | . | . | . | . | |
| StTotalArea2sd | LOGN | Identity | *F*_1,18_ = 1.51, *p* = 0.2355 | . | . | . | . | |
| StTotalArea3sd | EXPONENTIAL | Log | *F*_1,18_ = 0.07, *p* = 0.7932 | . | . | . | . | |
| Ac1SD peaks | N | Identity | *F*_1,18_ = 0.05, *p* = 0.8273 | . | . | . | . | |
| Ac2SD peaks | N | Identity | *F*_1,18_ = 0.65, *p* = 0.4293 | . | . | . | . | |
| Ac3SD peaks | IG | Reciprocal Square | *F*_1,18_ = 0.42, *p* = 0.5269 | . | . | . | . | |
| St1SD peaks | N | Identity | *F*_1,18_ = 2.66, *p* = 0.1204 | . | . | . | . | |
| St2SD peaks | LOGN | Identity | *F*_1,18_ = 3.04, *p* = 0.0983 | . | . | . | . | |
| St3SD peaks | IG | Identity | *F*_1,18_ = 2.34, *p* = 0.1438 | . | . | . | . | |
| Standing | IG | Reciprocal Square | *F*_1,44_ = 3.98, *p* = 0.0523 | *F*_1,44_ = 3.99, *p* = 0.0521 | *F*_1,44_ = 3.98, *p* = 0.0522 | *F*_1,44_ = 3.26, *p* = 0.0778 | . | |
| Walking | GAMMA | Log | *F*_1,47_ = 1.04, *p* = 0.3140 | *F*_1,47_ = 14.18, *p* = 0.0005 | *.* | *.* | . | |
| Lying | N | Identity | *F*_1,45_ = 1.15, *p* = 0.2890 | *F*_1,45_ = 5.46, *p* = 0.0240 | *F*_1,45_ = 5.88, *p* = 0.0194 | *F*_1,45_ = 5.32, *p* = 0.0257 | . | |
| Eating | GAMMA | Log | *F*_1,45_ = 0.13, *p* = 0.7153 | *F*_1,45_ = 1.65, *p* = 0.2049 | *F*_1,45_ = 3.39, *p* = 0.0724 | *F*_1,45_ = 4.08, *p* = 0.0494 | . | |
| Tail flicking | GAMMA | Log | *F*_1,45_ = 0.58, *p* = 0.4490 | *F*_1,45_ = 0.28, *p* = 0.5972 | *F*_1,45_ = 5.39, *p* = 0.0248 | *F*_1,45_ = 5.14, *p* = 0.0282 |  | |
| Foot stamping | GAMMA | Log | *F*_1,25_ = 0.01, *p* = 0.9184 | *F*_1,25_ = 0.88, *p* = 0.3566 | *.* | . | . | |
| Head turning | IG | Reciprocal Square | *F*_1,38_ = 2.78, *p* = 0.1037 | *F*_1,38_ = 6.05, *p* = 0.0186 | *F*_1,38_ = 5.07, *p* = 0.0302 | *F*_1,38_ = 4.19, *p* = 0.0475 |  | |
| Lesion licking | IG | Reciprocal Square | *F*_1,23_ = 3.30, *p* = 0.0823 | *F*_1,23_ = 6.25, *p* = 0.0200 | *F*_1,23_ = 4.70, *p* = 0.0407 | *F*_1,23_ = 3.82, *p* = 0.0628 | . | |

S3 Table. Changes in the strength and direction of the linear relationships between experimental variables. Sample sizes were not necessarily large enough to detect differences due to the treatment at the selected significance level (*α* = 0.05).

| **Table 1. Test of equality of correlations based on untransformed data** | | | | | | | | |
| --- | --- | --- | --- | --- | --- | --- | --- | --- |
| Variable_1_ | Variable_2_ | *n*_1_ | *z*_1_ | *n*_2_ | *z*_2_ | variance | *z* | *p*-value |
| Cortisol | RBC | 164 | -0.1016 | 160 | 0.14803 | 0.01258 | -2.2258 | 0.02603 |
| Cortisol | Temperature | 112 | -0.9458 | 106 | -0.6681 | 0.01888 | -2.0211 | 0.04327 |
| Hapto | Scrotal Circ. | 132 | 0.35131 | 120 | 0.63433 | 0.0163 | -2.2169 | 0.02663 |
| Infrared | RBC | 191 | -0.37 | 175 | -0.1179 | 0.01113 | -2.389 | 0.01689 |
| RBC | Eating | 30 | 0.43582 | 29 | -0.2079 | 0.0755 | 2.34281 | 0.01914 |
| SAA | SubP | 144 | -0.1575 | 131 | 0.19217 | 0.0149 | -2.8639 | 0.00418 |
| ScrotalCirc | Infrared | 132 | 0.43515 | 120 | 0.14601 | 0.0163 | 2.26479 | 0.02353 |
| SubP | Infrared | 192 | -0.278 | 172 | 0.14352 | 0.01121 | -3.9819 | 0.00007 |
| SubP | RBC | 191 | 0.5301 | 171 | 0.20026 | 0.01127 | 3.10686 | 0.00189 |
| Temperature | FootStamping | 30 | -0.194 | 30 | 0.35804 | 0.07407 | -2.0284 | 0.04252 |
| Temperature | TailFlicking | 30 | -0.2539 | 30 | 0.35059 | 0.07407 | -2.2209 | 0.02636 |
| WBC | FootStamping | 30 | -0.4438 | 29 | 0.82361 | 0.0755 | -4.6126 | <.0001 |
| WBC | TailFlicking | 30 | -0.1762 | 29 | 0.62764 | 0.0755 | -2.9254 | 0.00344 |
| Weight | FootStamping | 30 | 0.15482 | 30 | -0.4389 | 0.07407 | 2.18132 | 0.02916 |
| Weight | ScrotalCirc | 120 | 0.07087 | 106 | -0.2111 | 0.01826 | 2.08723 | 0.03687 |
| MealDuration | MealSize | 342 | 1.51496 | 318 | 1.30972 | 0.00612 | 2.62253 | 0.00873 |
| Standing | Lying | 287 | -9.2779 | 275 | -9.6128 | 0.0072 | 3.94738 | 0.00008 |

The data were rank-transformed using SAS PROC RANK by animal. Pearson’s product-moment correlation coefficients with Fisher’s *z* transformation were calculated based on the untransformed and rank-transformed data using SAS PROC CORR by treatment. The equality of correlations from the treatment groups (*ρ*_1_ and *ρ*_2_) were compared using Fisher’s *z* statistics (SAS PROC CORR). The null hypothesis *H*_0_: *ρ*_1_ = *ρ*_2_ was tested against the alternative *H*_1_: *ρ*_1_ ≠ *ρ*_2_. The *p*-value for testing *H*_0_ was derived by treating the difference *z*_1_ – *z*_2_ as a normal random variable with mean zero and variance 1 / (*n*_1_ – 3) + 1 / (*n*_2_ – 3), where *z*_1_ and *z*_2_ are Fisher’s *z* transformation of the sample correlations *r*_1_ and *r*_2_, respectively, and where *n*_1_ and *n*_2_ are the corresponding sample sizes.
